# The Complex Interplay between ERK1/2, TGFβ/Smad, and Jagged/Notch Signaling Pathways in the Regulation of Epithelial-Mesenchymal Transition in Retinal Pigment Epithelium Cells

**DOI:** 10.1371/journal.pone.0096365

**Published:** 2014-05-02

**Authors:** Xiaoyun Chen, Wei Xiao, Wencong Wang, Lixia Luo, Shaobi Ye, Yizhi Liu

**Affiliations:** State Key Laboratory of Ophthalmology, Zhongshan Ophthalmic Center, Sun Yat-sen University, Guangzhou, People's Republic of China; Florida International University, United States of America

## Abstract

Epithelial-mesenchymal transition (EMT) of retinal pigment epithelium (RPE) cells is a major pathologic change in the development of proliferative vitreoretinopathy (PVR), which leads to severe visual impairment. ERK1/2 pathway has been reported to play a key role in the carcinogenesis, cancer metastasis, and multiple fibrotic diseases. We hypothesized that ERK1/2 signaling could cross-interact with transforming growth factor β2 (TGFβ2)/Smad and Notch signaling pathways in the regulation of EMT in RPE cells. Here, we demonstrated that ERK1/2 signaling was activated in TGFβ2-induced EMT in human RPE cells, while blockade of the canonical TGFβ2/Smad2/3 signaling with SB431542 could not inhibit TGFβ2-induced the activation of ERK1/2. Meanwhile, blockade of ERK1/2 signaling with a specific MEK/ERK1/2 inhibitor U0126 strongly prevented TGFβ2-induced the downregulation of P-cadherin, and the upregulation of α-SMA, collagen type IV, N-cadherin and fibronectin in RPE cells. In addition, we also identified that blockade of ERK1/2 signaling could inhibit not only the canonical TGFβ/Smad signaling, but also the Jagged/Notch pathway. Finally, we found that blockade of Notch pathway with a specific inhibitor DAPT could inhibit TGFβ2-induced the activation of ERK1/2 pathway conversely. Therefore, our study provides evidence that ERK1/2 signaling can cross-interact with the canonical TGFβ/Smad and the Jagged/Notch signaling pathways in RPE cells EMT. ERK1/2 inhibitor may have therapeutic value in the prevention and treatment of PVR and other fibrotic diseases.

## Introduction

Proliferative vitreoretinopathy (PVR) is a severe complication of retinal detachment (RD) and ocular trauma, and the most common cause of surgical failure in the RD treatment. It occurs in 8–10% of patients with primary RD and 40–60% of patients with open-globe injury [1]. PVR is characterized by formation of pre- and sub-retinal fibrotic membranes, which reduce the flexibility of retina, and further result in retinal redetachment and difficulty in retinal reattachment [2]. Although advances in surgical techniques have reduced the PVR rate, it is still a great issue in RD and ocular trauma management.

The growing body of evidence shows that epithelial-mesenchymal transition (EMT) of retinal pigment epithelium (RPE) cells is a major pathologic change in the development of PVR [3], [4]. Retinal detachment and trauma give rise to the breakdown of the blood-retinal barrier (BRB), through which inflammatory cells, serum cytokines, and growth factors penetrate into the vitreous cavity and/or sub-retinal space [4]. This process allows the body to heal and repair the tissue damage. Several kinds of cells, including hyalocytes, retinal müller glial cells, fibroblasts and macrophages, are involved in this intraocular wound-healing response [5]. Of note, RPE cells are the most important contributor during this process [6]. RPE cells are mitotically inactive under physiological condition, however, the breakdown of BRB exposes RPE cells to a large amount of cytokines and growth factors in the vitreous. RPE cells are stimulated to proliferate, undergo EMT, and develop the ability to migrate towards the vitreous body or intraretinal layers through the retinal break. During this process, extracellular matrix (ECM) containing collagen and fibronectin are produced, and RPE cells transform into fibroblast-like cells constantly, which further results in the formation of pre- and sub-fibrous membranes [4]. The fibrotic membranes can contract and cause retinal wrinkling and distortion, leading to new retinal breaks formation and/or previously sealed breaks reopen, consequently resulting in severe visual impairment [7]. Therefore, agents capable of inhibiting the EMT of RPE cells may be of great therapeutic value in the prevention of PVR after retinal reattachment and trauma surgeries.

Transforming growth factor β (TGFβ) has been proven to be a multifunctional cytokine that induces EMT during embryonic development, wound healing, fibrotic diseases, and cancer metastasis [8], [9]. TGFβ2, the major TGFβ isoform in the posterior segment of the eye, is also the most important factor in PVR. Previous studies have reported that TGFβ2 is overexpressed in the vitreous and proliferative membranes from patients with PVR [10], [11]. TGFβ is known to transmit its signal through two main pathways: the canonical Smad-dependent pathway and the noncanonical Smad pathway. The canonical TGFβ/Smad signaling transmits signal via binding to two related transmembrane type I and type II receptors, which subsequently phosphorylate receptor-regulated Smad proteins-Smad2 and/or Smad3 [9]. Phosphorylated Smads partner with the common mediator Smad4, and then translocate to the nucleus and mediate gene transcription. In addition, other non-Smad signalings are also involved in TGFβ-induced EMT in different types of cells, including extracellular signal-regulated kinase (ERK) signaling, p38 mitogen-activated protein kinases (MAPKs), and phosphoinositide 3-kinase (PI3K)/AKT pathways [12]–[15]. Moreover, the noncanonical signals p38MAPK and PI3K/AKT pathways can crosstalk and integrate with the Smad pathway and mutually modulate each other [14], [16]. To make matters more complicated, these noncanonical TGFβ signals and the canonical Smad signaling can also be mediated by other signaling pathways, such as the Notch pathway [9]. In RPE cells, our previous study has demonstrated that ERK1/2 signaling pathway is activated by TGFβ2, however, the role of it has not been elaborated [17].

Despite the role of ERK1/2 signaling in EMT during cancer progressive and some fibrotic disorders has been studied, its function and interaction with other signaling pathways in ocular fibrotic diseases are still unknown. In this study, we identified that TGFβ2-induced the activation of ERK1/2 is independent of the canonical TGFβ/Smad pathway in human RPE cells. Blockade of ERK1/2 signaling with U0126 dramatically prevented TGFβ2-induced EMT through inhibiting not only the canonical Smad signaling pathway, but also the Jagged/Notch pathway. Moreover, we also found that ERK1/2 signaling induced by TGFβ2 can be mediated by the Notch pathway conversely. Collectively, these results suggest that ERK1/2 signaling can cross-interact with the canonical TGFβ/Smad and the Jagged/Notch signaling pathways in RPE cells EMT.

## Materials and Methods

### Reagents and antibodies

Recombinant human TGFβ2 and U0126 (a selective inhibitor of MEK 1 and MEK 2) were purchased from Cell Signaling (Danvers, MA). DAPT (an inhibitor of Notch receptor cleavage) and SB431542 (a specific inhibitor for TGFβ receptor type I/ALK5 kinase that phosphorylates Smad2/3) were purchased from Sigma-Aldrich (St Louis, MO). Antibodies against ERK1/2, p-ERK1/2, Jagged-1, Notch-3, p-Smad2, p-Smad3, Smad2, Smad3, horse anti-mouse and goat anti-rabbit horseradish peroxidase (HRP) conjugates secondary antibodies were purchased from Cell Signaling (Danvers, MA). Antibodies against β-actin, α-SMA, collagen type IV (Col IV), N-cadherin, P-cadherin and fibronectin (FN) were purchased from Abcam (Cambridge, UK).

### Cells culture and treatment

The human retinal pigment epithelial cell line ARPE-19 was kindly provided by Professor Fu Shang at the Laboratory for Nutrition and Vision Research (Boston, MA). It was obtained from ATCC [18]. The cells were cultured with Dulbecco's modified Eagle's medium (DMEM) containing 10% fetal bovine serum (FBS) at 37°C in a humidified atmosphere containing 5% CO_2_. The cells were dissociated with 0.25% trypsin-0.02% ethylenediaminetetravacetic acid (EDTA) solution after confluence.

Before treatment, RPE cells were grown to 80% confluence in six-well plates, and then incubated in serum-free medium for 12 h. Afterwards, various concentrations of U0126, SB431542 and DAPT were added to the cells 60 min prior to treatment with 5 ng/ml recombinant human TGFβ2. TGFβ2 was suspended in 4 mM HCl containing 0.5% BSA, and pharmacological inhibitors U0126, SB431542 and DAPT were dissolved in DMSO. Equivalent amounts of solvent were added to all control cultures.

### Real-time PCR analysis for gene expression

Total RNA was extracted from RPE cells using Trizol reagent according to the manufacture's instruction. Total RNA was treated with DNase I (Sigma-Aldrich, St Louis, MO) to remove genomic DNA, and then quantified by spectrophotometry. First-strand cDNA was synthesized with a reverse transcription kit (Takara; Siga, Japan) using conditions recommended by the manufacturer. For quantitative analysis of mRNA expression, SYBR PrimeScript RT-PCR kit (Takara, Siga, Japan) was used to amplify the target genes and the reactions were performed with the ABI Prism 7000 sequence detection system (Applied Biosystems, Foster City, CA). Glyceraldehyde 3-phosphate dehydrogenase (GAPDH) was used as an internal control. The primers used in the PCR reaction were described as follow: α-SMA, forward 5′-CCGACCGAATGCAGAAGGA-3′ and reverse 5′-ACAGAGTATTTGCGCTCCGAA-3′; Col IV, forward 5′-GCCCATGGTCAGGACTTG-3′ and reverse 5′-AAGGGCATGGTGCTGAACT -3′; N-Cadherin, forward 5′-ACAGTGGCCACCTACAAAGG-3′ and reverse 5′- CCGAGATGGGGTTGATAATG-3′; FN, forward 5′-GAGCTGCACATGTCTTGGGAAC-3′ and reverse 5′-GGAGCAAATGGCACCGAGATA-3′; Jagged-1, forward 5′-ACCAAGCAACAGATCCAAGC-3′ and reverse 5′-GAAACAGCTCGCTGATTGCT-3′; Notch-3, forward 5′-TGATGGCATGGATGTCAATGTG-3′ and reverse 5′-CAGTTGGCATTGGCTCCAGA-3′; Hes-1, forward 5′-CAACACGACACCGGATAAAC-3 and reverse 5′-TTCAGCTGGCTCAGACTTTC-3′; Hey-1, forward 5′-TGGATCACCTGAAAATGCTG-3′ and reverse 5′-TTGTTGAGATGCGAAACCAG-3′; GAPDH, forward 5′-GAGTCAACGGATTTGGTCGT-3′ and reverse 5′-AATGAAGGGGTCATTGATGG-3′.

### Western blot analysis for protein expression

For total protein extraction, cells were washed with cold PBS then lysed in 100 µl of RIPA buffer with protease inhibitor cocktail. The protein samples were mixed with 5×SDS sample buffer and then subjected to SDS-PAGE. After electroblotting onto the PVDF membranes, the membranes were blocked in 5% nonfat milk and incubated with different primary antibodies at 4°C overnight. The following day, the membranes were washed with 1×PBS containing 0.1% Tween-20 (PBST) for three times, and then incubated with HRP-conjugated secondary antibodies for 1 h at room temperature. The bands on the membranes were visualized using chemiluminescence detection reagents after washing three times with 1×PBST. Densitometic analysis was conducted by Image J software 1.41 (National Institutes of Health, Bethesda, MD). β-actin was used as loading control.

### Statistical analysis

Experiments presented in the figures are representative of three or more different repetitions. All data were expressed as mean ± standard error of the mean (SEM) and analyzed with SPSS 15.0 software (SPSS Inc., Chicago, IL). A standard student *t*-test was used for statistical analysis. A value of *p*<0.05 was considered statistically significant.

## Results

### Blockade of ERK1/2 pathway by U0126 prevents TGFβ2-induced EMT in RPE cells

**Figure 1:**
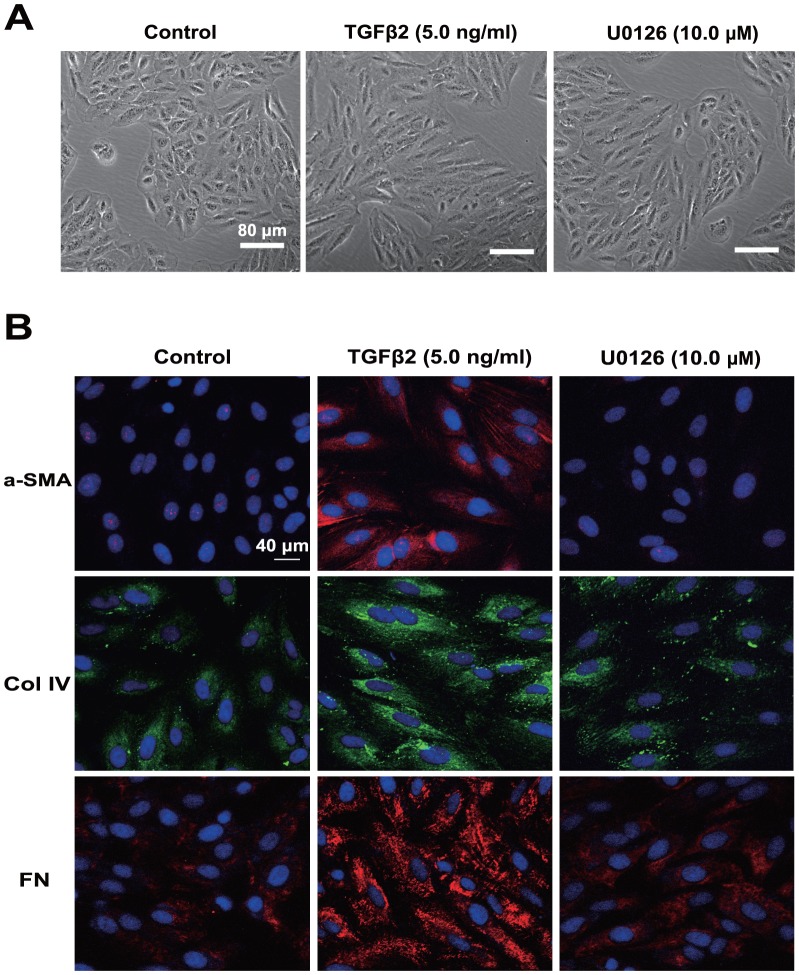
Blockade of ERK1/2 pathway by U0126 prevents TGFβ2-induced EMT in RPE cells. RPE cells were cultured in the absence or presence of TGFβ2 (5 ng/ml) with U0126 (10.0 µM) or DMSO for 24 h. (A) Cell morphology was examined using phase contrast microscope under ×100 magnification. Scale bar = 80 µm. (B) Immunofluorescence analysis of α-SMA (red), Col IV (green), and FN (red) using confocal microscopy. Representative images are shown (scale bar = 40 µm).

**Figure 2:**
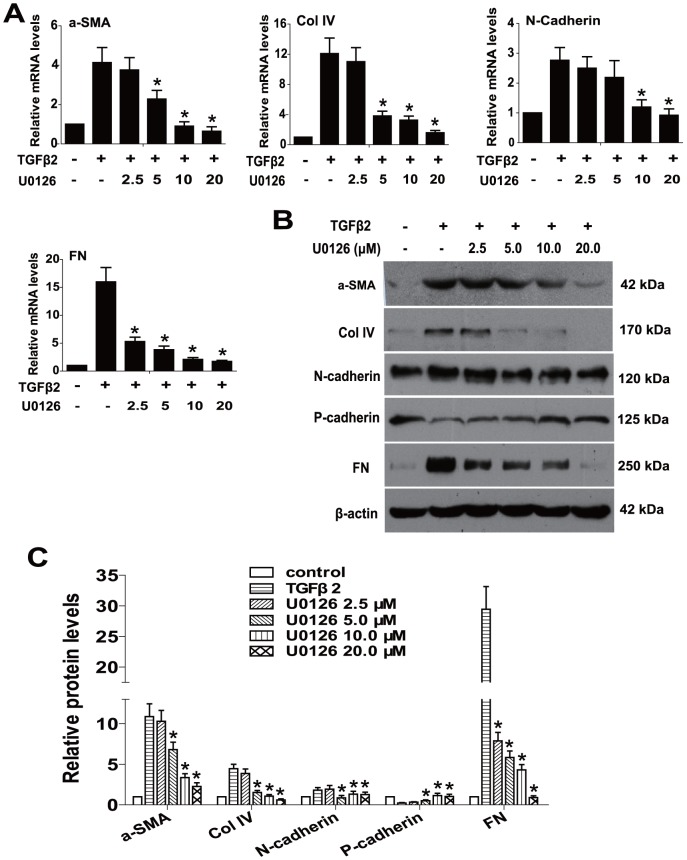
U0126 prevents TGFβ2-induced EMT through upregulating epithelial cell marker and downregulating EMT markers expression. RPE cells were cultured in the absence or presence of TGFβ2 (5 ng/ml) with U0126 (2.5, 5.0, 10.0, 20.0 µM) or DMSO for 24 h. (A) The mRNA expression levels of α-SMA, Col IV, N-cadherin and FN were determined by real-time quantitative PCR. Gene expression levels were normalized to the GAPDH control. *, *P<*0.05 *v*. TGFβ2 treated with DMSO group. (B) The protein expression levels of α-SMA, Col IV, N-cadherin, P-cadherin and FN were detected by western blot. (C) Quantification of protein levels from three independent experiments. *, *P<*0.05 *v*. TGFβ2 treated with DMSO group.

To explore whether blockade of ERK1/2 pathway could prevent TGFβ2-induced EMT in RPE cells, U0126 (a selective inhibitor of MEK1 and MEK2) was used. Cell morphology, epithelial cell marker P-cadherin, and EMT markers such as α-SMA, Col IV, N-cadherin and FN were investigated. As showed in Fig. 1A, treatment of RPE cells with TGFβ2 resulted in obvious changes in cell morphology, presenting as marked transition from an epithelial to a more mesenchymal phenotype. In accordance with that, immunofluorescence staining of α-SMA, FN and Col IV were enhanced dramatically (Fig.1B). In addition, the real-time PCR results showed that the expression of α-SMA, Col IV, N-cadherin and FN were upregulated about 4.1-, 12.1-, 2.8- and 16.9-fold in TGFβ2-induced RPE cells (Fig.2A). At the same time, the western blot results also revealed that the expression of α-SMA, Col IV, N-cadherin and FN were increased obviously, while the expression of P-cadherin was decreased after TGFβ2 treatment (Fig.2B and C). Intriguingly, U0126 treatment completely abrogated the morphological changes of RPE cells, as well as the upregulation of α-SMA, Col IV, N-cadherin and FN, and the downregulation of P-cadherin (Fig. 1 and 2:*, *P<*0.05 *v*. TGFβ2 treated with DMSO group). Maximum effect of U0126 was observed at a concentration of 20.0 µM, however, there was no obvious difference between 10.0 and 20.0 µM. Hence, 10.0 µM of U0126 was used for the following experiments. Taken together, these data indicate that blockade of ERK1/2 pathway by U0126 can completely attenuate TGFβ2-induced EMT in RPE cells.

### TGFβ2-induced ERK1/2 activation is independent of TGFβ/Smad pathway

**Figure 3:**
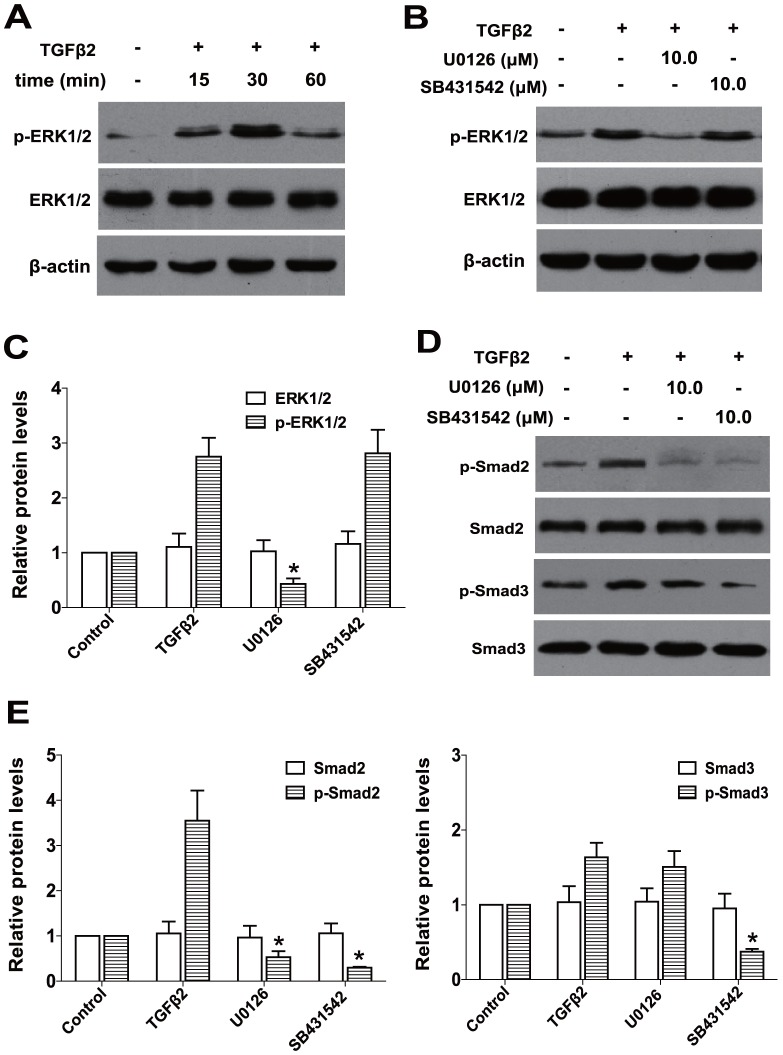
TGFβ2-induced ERK1/2 activation is independent of TGFβ/Smad pathway and U0126 inhibits TGFβ2-induced phosphorylation of Smad2. (A) RPE cells were cultured in the absence or presence of TGFβ2 for 15min, 30 min and 60 min, the expression of p-ERK1/2 and ERK1/2 were determined by western blot. (B) RPE cells were cultured in the absence or presence of TGFβ2 with U0126 (10.0 µM) or SB431542 (10.0 µM) for 30 min, the expression of p-ERK1/2 and ERK1/2 were determined by western blot. (C) Quantification of protein levels from three independent experiments. *, *P<*0.05 *v*. TGFβ2 treated with DMSO group. (D) The phosphorylation and the total levels of Smad2 and Smad3 were detected by western blot after 60 min treatment. (E) Quantification of protein levels from three independent experiments. *, *P<*0.05 *v*. TGFβ2 treated with DMSO group.

Firstly, we examined whether ERK1/2 could be activated by the treatment with TGFβ2 in RPE cells. As shown in Fig.3A, when RPE cells were stimulated with TGFβ2 for 15 min, ERK1/2 was activated through phosphorylation and reached maximum after 30 min, but this activation was rapidly deactivated over 60 min. Next, to determine whether the canonical Smad signaling is required for the activation of ERK1/2 pathway by TGFβ2, we used SB431542, a specific inhibitor for TGFβ receptor type I/ALK5 kinase that phosphorylates Smad2/3. As illustrated in Fig.3B and C, co-treatment with U0126 could absolutely inhibit TGFβ2-induced phosphorylation of ERK1/2, however, SB431542 had no effect on the phosphorylation of ERK1/2 (Fig.3B and C:*, *P<*0.05 *v*. TGFβ2 treated with DMSO group). These results suggest that TGFβ2-induced ERK1/2 activation is independent of the canonical TGFβ/Smad pathway in RPE cells.

### U0126 mediates the canonical TGFβ/Smad signaling via suppressing the phosphorylation of Smad2

To clarify whether there is a crosstalk between the ERK1/2 signaling and the canonical TGFβ/Smad pathway, the impact of U0126 on the activation of receptor-regulated Smad proteins Smad2 and Smad3 were investigated. As shown in Fig.3D and E, TGFβ2 alone induced significant phosphorylation of Smad2 and Smad3 after treatment for 60 min, co-treatment with SB431542 could completely inhibit TGFβ2-induced phosphorylation of Smad2 and Smad3, whereas U0126 treatment inhibited the phosphorylation of Smad2, but had no effect on the phosphorylation of Smad3 (Fig.3D and E:*, *P<*0.05 *v*. TGFβ2 treated with DMSO group). These results indicate that U0126 can inhibit the canonical TGFβ2/Smad signaling through suppressing the phosphorylation of Smad2. Thus, there is a crosstalk between the ERK1/2 signaling and the canonical TGFβ2/Smad signaling pathway in RPE cells.

### U0126 prevents TGFβ2-induced EMT partly through suppressing the Jagged/Notch pathway

**Figure 4:**
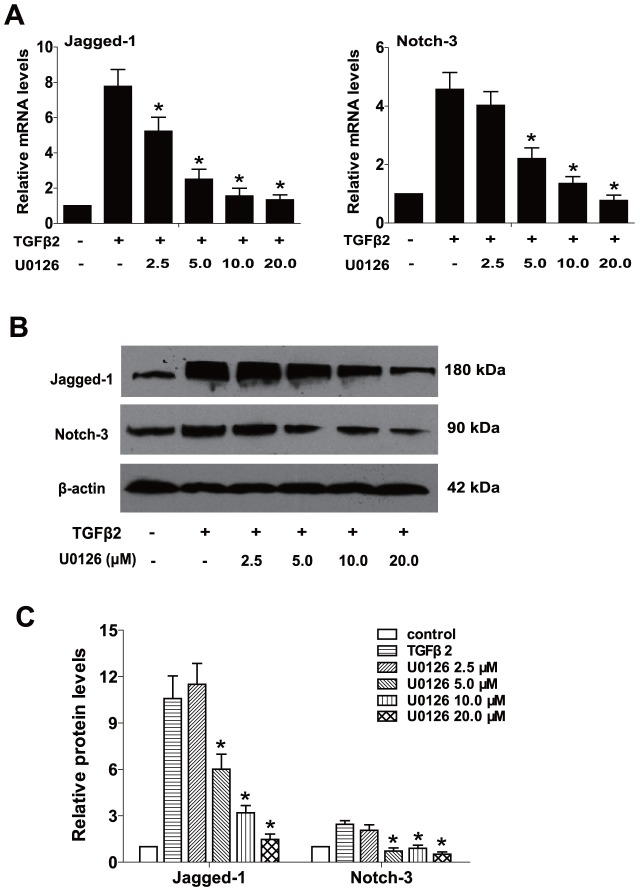
U0126 prevents TGFβ2-induced EMT via suppressing the Notch pathway. (A) RPE cells were treated with TGFβ2 in the presence of U0126 (2.5, 5.0, 10.0, 20.0 µM) or DMSO for 24 h, the mRNA expression levels of Jagged-1and Notch-3 were detected by real-time PCR. Gene levels were normalized to control GAPDH. *, *P<*0.05 *v*. TGFβ2 treated with DMSO group. (B) The protein expression levels of Jagged-1and Notch-3 were detected by western blot. (C) Quantification of protein levels from three independent experiments. *, *P<*0.05 *v*. TGFβ2 treated with DMSO group.

**Figure 5:**
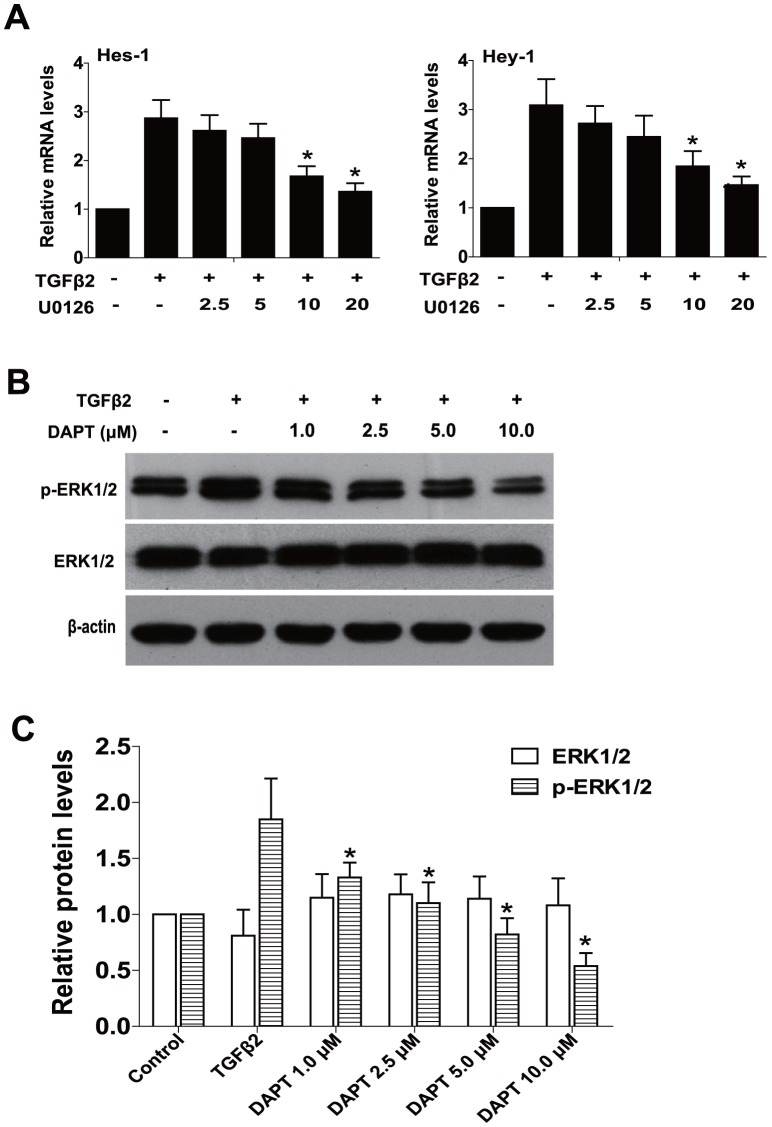
U0126 suppresses Notch target genes expression and blockade of Notch pathway inhibits ERK1/2 pathway activation. (A) RPE cells were treated with TGFβ2 in the presence of U0126 (2.5, 5.0, 10.0, 20.0 µM) or DMSO for 24 h, the mRNA expression levels of Notch target genes Hes-1and Hey-1 were detected by real-time PCR. Gene levels were normalized to control GAPDH. *, *P<*0.05 *v*. TGFβ2 treated with DMSO group. (B) RPE cells were cultured in the absence or presence of TGFβ2 with DAPT (1.0, 2.5, 5.0, 10.0 µM) or DMSO for 30 min, the expression of p-ERK1/2 and ERK1/2 were determined by western blot. (C) Quantification of protein levels from three independent experiments. *, *P<*0.05 *v*. TGFβ2 treated with DMSO group.

Increasingly evidence suggests that the Notch signaling pathway is a crucial regulator in the induction of EMT during embryonic development, fibrotic diseases and cancer metastasis [19]. Our previous study also reported that Jagged/Notch pathway is activated via the canonical TGFβ2/Smad signaling during TGFβ2-induced EMT of human RPE cells, while blockade of Notch pathway inhibits TGFβ2-induced EMT effectively [20]. Therefore, we next explored whether blockade of ERK1/2 signaling with U0126 could suppress the Notch signaling activated by TGFβ2, and then further to suppress RPE cells EMT. As shown in Fig. 4, TGFβ2 treatment alone markedly increased the expression of Jagged-1 and Notch-3 at mRNA and protein levels, while U0126 treatment could completely reverse the upregulation of Jagged-1 and Notch-3 (Fig. 4, *, *P<*0.05 *v*. TGFβ2 treated with DMSO group). Moreover, treatment with U0126 could also downregulate TGFβ2-induced Notch target genes Hes-1 and Hey-1 expression (Fig. 5A, *, *P<*0.05 *v*. TGFβ2 treated with DMSO group). These results indicate that U0126 abrogates TGFβ2-induced EMT partly through suppressing the Jagged/Notch pathway. In other words, the noncanonical ERK1/2 signaling also contributes to TGFβ2-induced the activation of Notch pathway in RPE cells.

### The noncanonical TGFβ/ERK1/2 signaling can be mediated by the Notch pathway

On the contrary, whether blockade of Notch signaling can mediate the ERK1/2 signaling pathway activated by TGFβ2 remains unknown. As we expected, inactivation of Notch pathway by DAPT could distinctly inhibit TGFβ2-induced the activation of ERK1/2 pathway in a concentration-dependent manner in RPE cells (Fig. 5B and C: *, *P<*0.05 *v*. TGFβ2 treated with DMSO group). These data suggest that the noncanonical TGFβ/ERK1/2 signaling can also be mediated by the Notch pathway reversely. This also implies that there is a crosstalk between the ERK1/2 signaling and the Notch pathway in RPE cells EMT.

## Discussion

Emerging evidence has proven that the development of PVR largely attributes to the EMT of RPE cells in response to a variety of cytokines, typically TGFβ2. Activation of ERK1/2 pathway is frequently observed and plays a major role in the carcinogenesis and metastasis of cancers, and various fibrotic diseases [21]–[23]. In the present study, we examined the role of the ERK1/2 pathway in TGFβ2-induced EMT in human RPE cells, with a focus on the interaction of ERK1/2 signaling with the canonical TGFβ2/Smad and the Jagged/Notch pathways. We demonstrated that the activation of ERK1/2 signaling by TGFβ2 is independent of the canonical TGFβ2/Smad signaling in RPE cells. Moreover, inactivation of ERK1/2 signaling with U0126 completely abrogates TGFβ2-induced EMT through inhibiting not only the canonical Smad signaling pathway, but also the Jagged/Notch pathway. Finally, we also identified that the noncanonical TGFβ/ERK1/2 signaling can be mediated by the Notch pathway in RPE cells EMT. Hence, our study suggests that ERK1/2 signaling can cross-interact with the canonical TGFβ/Smad and the Jagged/Notch signaling pathways in RPE cells EMT.

TGFβ signaling occupies a central position in the signaling networks that control EMT. It is not limited to the canonical Smad signaling, it also can mediate through the non-Smad signaling pathways. Recently, various studies have demonstrated that the activation of ERK1/2 signaling in response to TGFβ [14], [24], [25]. The activation of ERK1/2 signaling enhances TGFβ-induced EMT, accompanied by the morphological changes and the up-regulation of EMT markers and ECM components. Blocking the function of MEK1/2 using a special inhibitor results in the inactivation of ERK1/2 and the inhibition of TGFβ-induced EMT [26]. Our data also showed that ERK1/2 is activated by TGFβ2 stimulation, however, SB431542, a specific inhibitor for TGFβ/Smad2/3 signaling transduction, cannot inhibit the activation of ERK1/2. These results suggest that TGFβ2-induced ERK1/2 activation is independent of the canonical TGFβ/Smad pathway in RPE cells. Furthermore, blockade of ERK1/2 signaling also dramatically prevents the morphological changes of RPE cells and the up-regulation of EMT markers induced by TGFβ2. Collectively, these data suggest that ERK1/2 signaling pathway is also a crucial regulator for TGFβ induction of EMT in RPE cells, and ERK1/2 inhibitor can be useful for abolishing EMT phenotype.

Recent evidence shows that the noncanonical Smad signaling, such as the p38MAPK and the PI3K/AKT pathways, can crosstalk and interact with the canonical TGFβ/Smad signaling during EMT process [14], [16]. To examine whether there is a crosstalk between the noncanonical TGFβ/ERK1/2 signaling and the canonical TGFβ/Smad signaling, the effect of U0126 on the activation of Smad2 and Smad3 induced by TGFβ2 were examined. We found that U0126 is able to inhibit the phosphorylation of Smad2 induced by TGFβ2, but has no effect on the phosphorylation of Smad3 in RPE cells. These results imply that U0126 can mediate the canonical TGFβ/Smad signaling through suppressing the phosphorylation of Smad2. In other words, there is a crosstalk between the noncanonical TGFβ/ERK1/2 signaling and the canonical TGFβ/Smad signaling in RPE cells EMT.

Emerging evidence has verified that the Notch signaling pathway is a key regulator in the induction of EMT during embryonic development, fibrotic diseases and cancer metastasis [19]. The Jagged/Notch signaling is elevated in a large range of fibrotic diseases developed in the liver, kidney and lung [27]. Furthermore, our previous study also found that the elements of the Notch signaling pathway, including Jagged-1, Notch-3, Hes-1, and Hey-1 are upregulated in TGFβ2-stimulated EMT in human RPE cells, while blockade of Notch pathway with DAPT absolutely reverses TGFβ2-induced EMT. The Notch signaling pathway is activated via the canonical TGFβ2/Smad signaling in RPE cells EMT [20]. In this study, we demonstrated that inactivation of ERK1/2 signaling can not only reduce TGFβ2-induced the upregulation of Jagged-1 and Notch-3, but also reduce Notch target genes Hes-1 and Hey-1 expression. These results suggest that U0126 abrogates TGFβ2-induced EMT partly via downregulating the Jagged/Notch pathway. Besides the canonical TGFβ2/Smad signaling, the noncanonical ERK1/2 signaling is also involved in TGFβ2-induced the activation of Notch pathway in RPE cells. In addition, we also found that blockade of Notch pathway with DAPT can inhibit TGFβ2-induced the activation of ERK1/2 pathway. This implies the noncanonical TGFβ/ERK1/2 signaling can also be mediated by the Notch pathway in RPE cells EMT. Taken together, these data indicate that there is also a crosstalk between the ERK1/2 signaling and the Jagged/Notch pathway in RPE cells EMT.

To sum up, our study provides evidence that TGFβ2-induced the activation of ERK1/2 is independent of the canonical TGFβ/Smad pathway in human RPE cells. Blockade of ERK1/2 signaling with U0126 dramatically reverses TGFβ2-induced EMT in RPE cells. Moreover, inactivation of ERK1/2 signaling can inhibit not only the canonical Smad signaling pathway, but also the Jagged/Notch pathway. Conversely, the noncanonical TGFβ/ERK1/2 signaling can also be mediated by the Notch pathway. Therefore, our results indicate that ERK1/2 signaling can cross-interact with the canonical TGFβ/Smad and the Jagged/Notch signaling pathways in RPE cells EMT. ERK inhibitor may have therapeutic value in the prevention and treatment of PVR and other fibrotic diseases.
